# Endochitinase Overexpression in Transgenic Tomato Plants Improves Resistance to Phytopathogens

**DOI:** 10.1002/biot.70297

**Published:** 2026-08-20

**Authors:** Lucas José de Sousa, Ana Carolina Mendes Bezerra, Ivonaldo Reis Santos, Ana Clara Muniz Martins Vilela, Osmundo Brilhante de Oliveira Neto, Glaucia Barbosa Cabral, Alice Maria Quezado‐Duval, Pedro Ricardo Vieira Hamann, Eliane Ferreira Noronha, Luiz Eduardo Bassay Blum, Angela Mehta

**Affiliations:** ^1^ Embrapa Recursos Genéticos e Biotecnologia Brasília Brazil; ^2^ Departamento De Fitopatologia Universidade De Brasília Brasília Brazil; ^3^ Embrapa Hortaliças Gama Brazil; ^4^ Departamento De Biologia Celular Universidade De Brasília Brasília Brazil

**Keywords:** *Agrobacterium tumefaciens*, broad‐spectrum resistance, Endochitinase, PR proteins, transgenic

## Abstract

Engineering broad‐spectrum disease resistance remains a major challenge in crop improvement because most resistance genes are effective against a limited range of pathogens. Pathogenesis‐related (PR) proteins, particularly endochitinases, are promising targets for enhancing plant immunity due to their antimicrobial activity and ability to activate defense responses. Here, we generated transgenic tomato (*Solanum lycopersicum* L.) plants overexpressing an endochitinase gene to assess their potential for enhanced resistance against bacterial and fungal pathogens. Three independent overexpression lines and the wild type were evaluated for transgene expression, chitinase activity, defense‐related gene expression, and resistance after inoculation with *Xanthomonas euvesicatoria* pv. *perforans* (Xep) and *Sclerotinia sclerotiorum*. The transgene was strongly expressed in all transgenic lines, whereas chitinase activity increased by up to fivefold compared with the wild type. Endochitinase overexpression activated defense responses, with marked induction of *PR1* expression, reaching a maximum increase of over 480‐fold, and enhanced expression of defense‐associated genes, including *SlACS* and *SlACO*. Compared with the wild type, the most responsive transgenic lines showed reductions of up to 79.7% in bacterial spot severity and 85.6% in white mold severity. These findings demonstrate that endochitinase overexpression activates defense signaling and improves broad‐spectrum resistance, representing a promising strategy for developing disease‐resistant tomato cultivars.

## Graphical Abstract and Lay Summary

Transgenic tomato plants overexpressing an endochitinase gene showed confirmed transgene expression and enhanced resistance. Upon plants inoculations with *Xanthomonas euvesicatoria* pv. *perforans* and *Sclerotinia sclerotiorum*, disease severity was significantly reduced compared to wild‐type plants, demonstrating broad‐spectrum disease resistance.

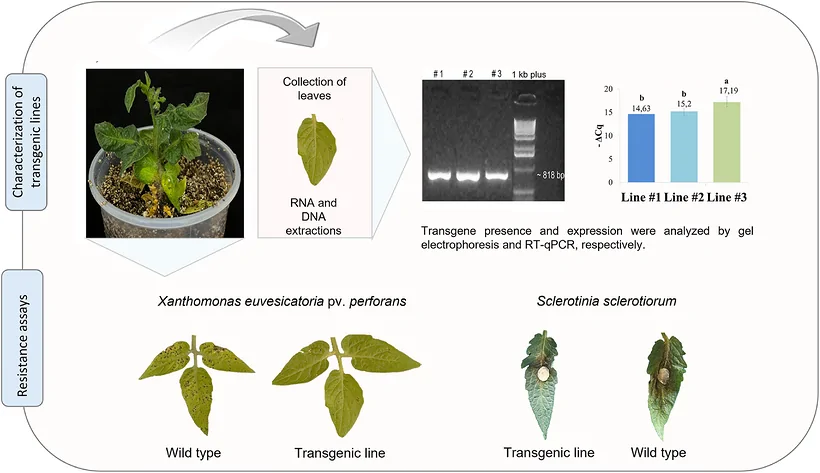

## 1 Introduction

Tomato (*Solanum lycopersicum* L.) is one of the most economically important crops worldwide, and yet it faces significant yield losses due to attacks from phytopathogens, such as *Xanthomonas euvesicatoria* pv. *perforans* (Xep) and *Sclerotinia sclerotiorum*, which cause bacterial spot and white mold, respectively [1, 2, 3]. Bacterial spot control strategies have been following the same recommendations over the last 40 years, primarily relying on copper‐based products. Throughout this period, the bacterium has developed mechanisms to overcome copper resistance [4], and currently, the cost of these products may not yield significant financial returns for growers [5]. Moreover, this approach has had negative impacts on non‐target species [6]. As for Xep, the primary methods for controlling *S. sclerotiorum* also still rely on cultural practices, as well as chemical and biological control, and currently, there are no resistant tomato cultivars available [7].

Resistance genes associated with hypersensitive responses have been identified and mapped in tomato germplasm specific to races T3 and T4 of Xep. Additionally, quantitative loci (QTL) have been mapped on chromosome 11 in three genotypes that exhibit broad‐spectrum resistance to the *Xanthomonas* complex [8]. Although broad resistance to the *Xanthomonas* complex has been achieved in tomato plants, the use of resistant cultivars in the field is still lacking, and resistance to multiple diseases remains limited, requiring further research.

Understanding the behavior of resistant and susceptible plants during interactions with pathogens is crucial for developing resistant cultivars [9]. Research has shown that resistant plants can recognize pathogen effectors, triggering signaling cascades in the salicylic acid (SA) pathway. This activation leads to the production of Pathogenesis‐Related Proteins (PR Proteins) and the establishment of Systemic Acquired Resistance (SAR) [10, 11]. In contrast, susceptible plants exhibit an increase in proteins associated with abscisic acid (ABA) synthesis. The ABA pathway antagonizes the SA pathway, resulting in lower expression of PR proteins in susceptible plants compared to their resistant counterparts [12, 13].

PR proteins are important components of plant defense and have been widely used to enhance resistance against phytopathogens [14]. These proteins are constitutively expressed at low levels and are strongly induced by biotic and abiotic stimuli, acting as mediators of local and systemic defense responses [15]. Among them, chitinases constitute an important group due to their ability to hydrolyze chitin, a major structural component of fungal cell walls [16, 17]. Although traditionally associated with defense against fungi and insects, chitinases can also interact with carbohydrates present in bacterial cell walls, promoting the agglutination of Gram‐negative bacteria [18]. In addition, PR protein overexpression may activate the expression of other defense‐related genes and signaling pathways, enhancing protection against multiple phytopathogens [19].

Previous proteomics and overexpression studies in *Arabidopsis thaliana* have shown that the endochitinase gene (*CHI‐B4 like*) enhances the resistance of *Brassica oleracea* plants to *Xanthomonas campestris* [20]. Building on this finding, the present study aimed to evaluate the effect of endochitinase overexpression in tomato plants, specifically investigating their resistance to the bacterium Xep and the fungus *S. sclerotiorum*.

## 2 Results and Discussion

Our efforts to enhance the resistance of tomato plants have focused on the overexpression of a PR protein, specifically a chitinase, due to its multifunctional role in plant defense against multiple pathogens. In a proteomic study, the endochitinase gene (*CHI‐B4 like*) was first identified in a resistant variety of cabbage (*B. oleracea*) that showed resistance to *X. campestris* pv. *campestris*. This gene was subsequently functionally validated through overexpression in *A. thaliana*, showing resistance against the pathogen [20]. The heterologous expression of chitinases has been employed in various plant species to enhance resistance, particularly against fungal pathogens such as *S. sclerotiorum* [21] and *Erysiphe cichoracearum* [19]. These genes have also been employed for nematode resistance [22]; however, there is limited evidence regarding the effectiveness of chitinases in providing resistance to phytopathogenic bacteria [23].

**FIGURE 1:**
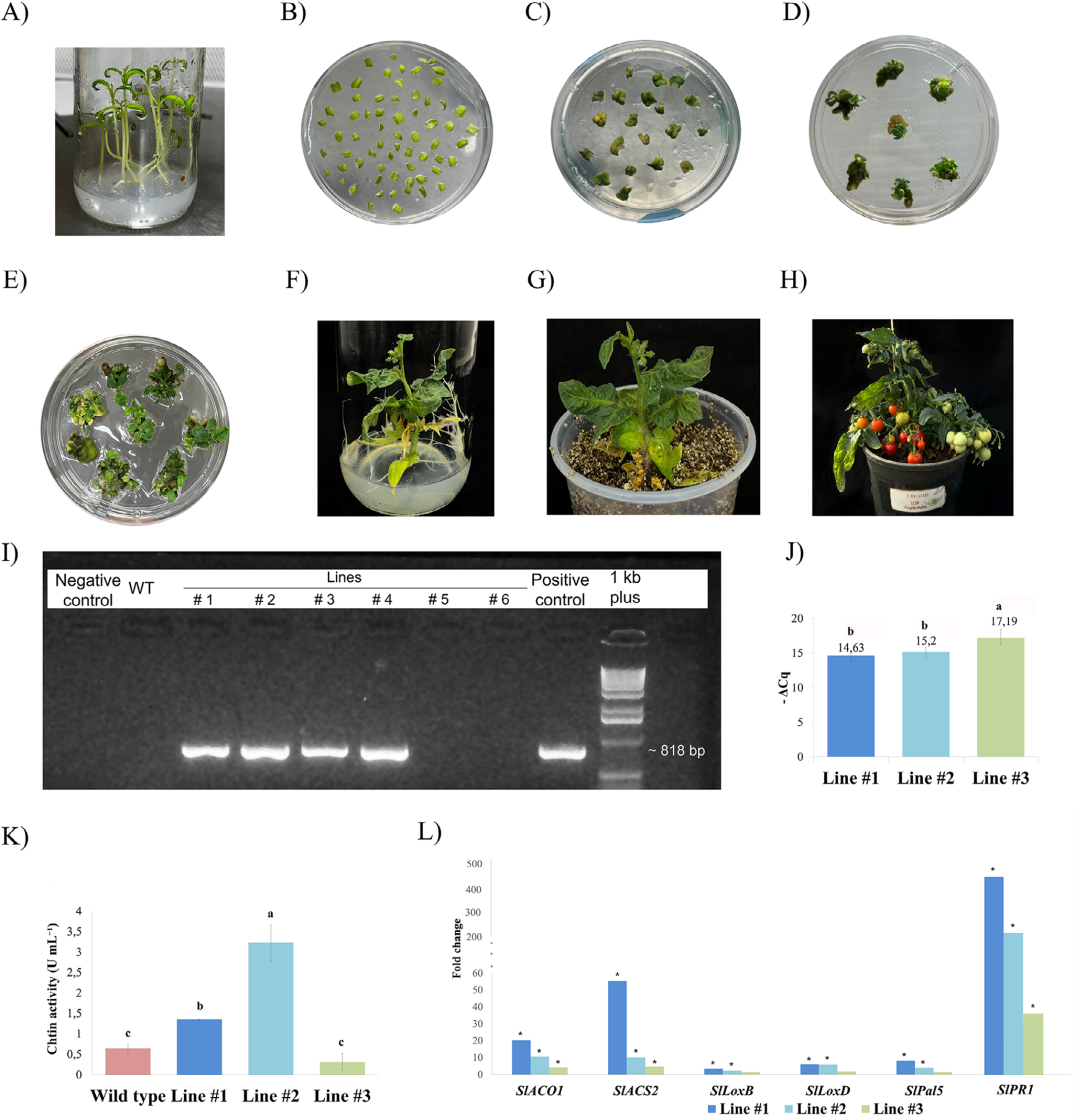
Steps for the regeneration of transgenic tomato plants expressing the *CHI‐B4 like* gene. (A) Germinating seedlings of MicroTom; (B) Cotyledon explants on solid cocultivation medium with *A. tumefaciens*; (C–E) Explants after cocultivation on bud induction medium under kanamycin selection; (F) Rooted regenerated plant (G) Acclimatized plant in the greenhouse; (H) Plant (T0) producing flowers and fruits in the greenhouse; (I) Identification of positive plant lines containing the *CHI‐B4 like* gene; (J) Expression levels of the *CHI‐B4 like* transgene. Data represent the mean ± SD of three biological replicates. Different letters indicate significant differences among lines (*p* < 0.05). (K) Endochitinase activity against colloidal chitin in wild‐type and transgenic lines. Data represent the mean ± SD. Different letters indicate significant differences (*p* < 0.05). (L) Relative expression of defense genes in leaves of transgenic lines. Asterisks indicate significant differences compared with the wild‐type (*p* ≤ 0.05).

Our overexpression experiments, conducted through stable transformation mediated by *Agrobacterium tumefaciens*, resulted in the regeneration of Micro‐Tom tomato plants exhibiting shoot, root, flower, fruit, and seed development comparable to that of non‐transformed plants (Figure 1A–H). Two co‐cultures comprising 45 seedlings were used to generate a total of 360 explants. From these explants, 18 regenerated plants were obtained, of which four lines were confirmed as positive for the CHI‐B4 like transgene by PCR analysis (Figure 1I). Accordingly, the regeneration rate was 5%, the transformation efficiency was 1.11%, and the positive rate among regenerated plants was 22.22%. Sequence alignment of these four transgenic lines revealed 100% similarity to the *CHI‐B4 like* gene sequence from *B. oleracea*, indicating successful integration of the transgene into the tomato plant genome (Figure S1).

The quantification of *CHI‐B4 like* transgene expression in transgenic lines (#1, #2, and #3) revealed that Line #3 exhibited the highest expression level (−ΔCq = 17.19 ± 1.26) (Figure 1J), followed by Lines #2 (−ΔCq = 15.20 ± 0.60) and #1 (−ΔCq = 14.63 ± 0.09). As expected, no expression was detected in wild‐type plants (Cq ≥ 40). Statistical analysis indicated that Line #3 showed a significantly higher expression compared to Line #1 and Line #2 (*p* < 0.001), whereas no significant difference was observed between Lines #1 and #2 (*p* = 0.12). Chitinase activity differed significantly among the evaluated lines (Figure 1K). Line #2 exhibited the highest enzymatic activity (3.23 ± 0.44 U mL^−^^1^), representing a fivefold increase compared with the wild type, followed by Line #1 (1.35 ± 0.01 U mL^−^^1^), which showed a twofold increase in activity. In contrast, the wild type (0.65 ± 0.12 U mL^−^^1^) and Line #3 (0.32 ± 0.20 U mL^−^^1^) showed lower enzyme activity and did not differ significantly from each other. Overall, the increased chitinase activity observed in Lines #1 and #2 is consistent with the expression of the *CHI‐B4 like* transgene in these lines. The relative expression data of defense‐related genes revealed that Line #1 exhibited the highest transcript accumulation among all evaluated genes, with a particularly pronounced increase in *Pathogenesis‐Related 1* (*SlPR1*) gene expression (489.513) (Figure 1L), a well‐established marker associated with SA‐mediated defense responses. This elevated *SlPR1* expression suggests a strong activation of SA‐dependent defense signaling in this line. Additionally, the *1‐Aminocyclopropane‐1‐Carboxylate Synthase* (*SlACS*) (55.3) and *1‐Aminocyclopropane‐1‐Carboxylate Oxidase* (*SlACO*) (20.146) genes showed increased expression, indicating enhanced activation of ethylene biosynthesis‐related pathways. Line #2 displayed an intermediate expression profile, with *SlPR1* transcript accumulation remaining considerably elevated (225.766), while *SlACO* (10.477) and *SlACS* (10.085) showed moderate expression levels. Conversely, although most of the analyzed genes were up‐regulated in Line #3, lower expression levels were observed compared to the others lines. The expression of *SlPR1* (35.262) was substantially reduced, and the other defense‐related genes also exhibited consistently lower transcript levels, particularly *Lipoxygenase B* (*SlLoxB*) (1.265) and *Phenylalanine ammonia‐lyase 5* (*SlPAL5*) (1.308).

**FIGURE 2:**
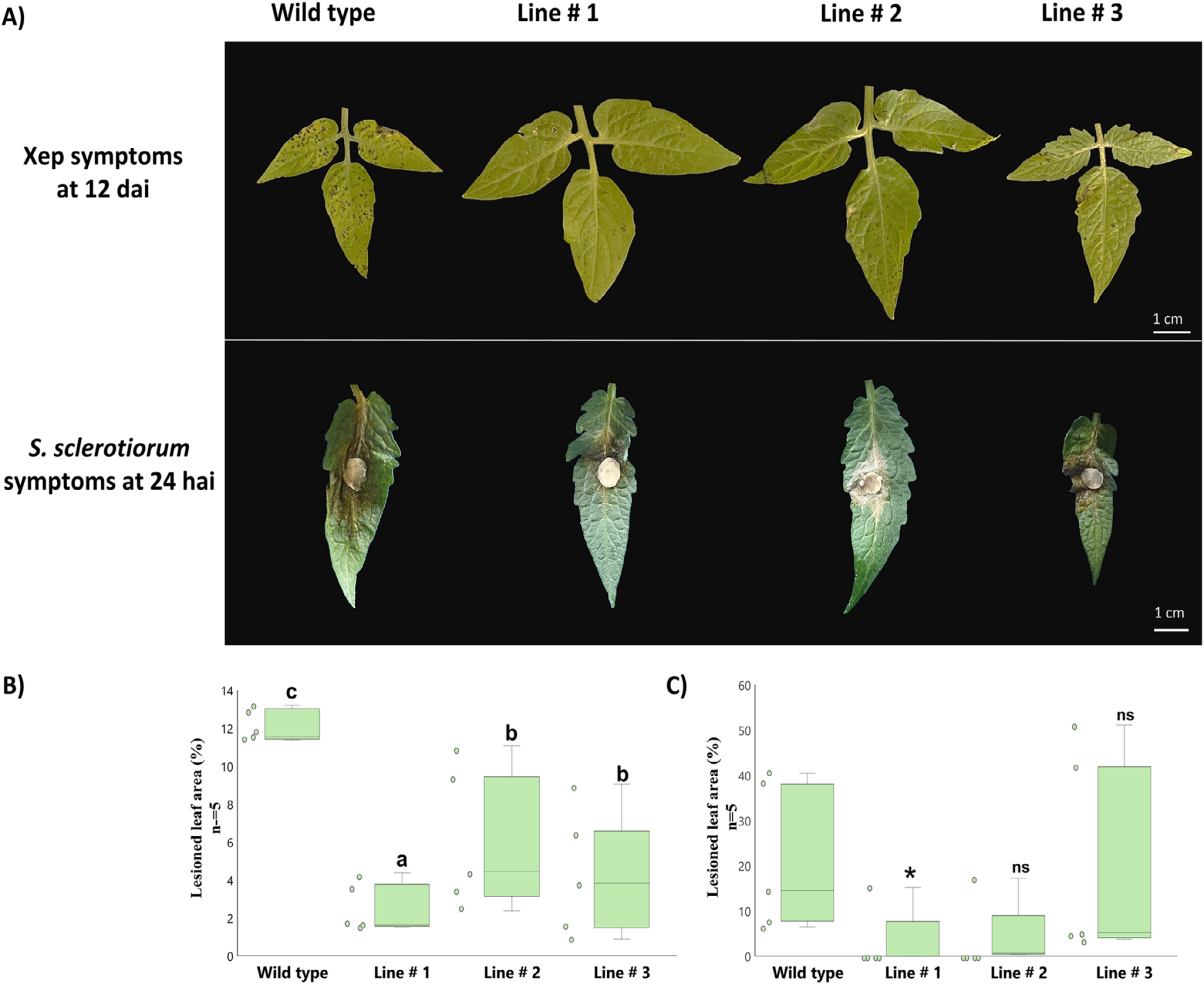
Disease severity caused by infection with *Xanthomonas euvesicatoria* pv. *perforans* (Xep) and *Sclerotinia sclerotiorum*. (A) Representative symptoms observed at 12 days after inoculation (dai) with Xep and at 24 h after inoculation (hai) with *S. sclerotiorum*. (B–C). Quantification of the percentage of lesioned leaf area caused by Xep and *S. sclerotiorum*, respectively. Individual biological replicates (*n* = 5) are shown beside each bar to illustrate data distribution within treatments. Asterisks (*) indicate statistically significant differences compared with wild‐type plants according to Dunn's multiple comparison test (*p* ≤ 0.05), while different letters above the bars indicate statistically significant differences among transgenic lines (*p* ≤ 0.05). Lines sharing the same letter are not significantly different.

To determine whether the observed molecular responses translated into enhanced disease resistance, the transgenic lines were challenged with two taxonomically distinct pathogens, the bacterium Xep and the fungus *S. sclerotiorum*. Indeed, this could be observed in our plant resistance analysis. Typical symptoms of bacterial spot caused by Xep were observed at 12 days after inoculation (dai), characterized by water‐soaked areas on the leaves that progressed to necrosis and the appearance of small lesions. However, significant differences in disease severity (p ≤ 0.05) were observed among the evaluated lines, with transgenic plants showing milder symptoms compared with wild‐type plants (Figures 2A,B and S2). Based on lesioned area measurements, transgenic Lines #1, #2, and #3 exhibited reductions of approximately 79.7%, 51.1%, and 67.1% in disease severity, respectively, compared to wild‐type plants. Similarly, transgenic plants displayed greater tolerance to the initial development of *S. sclerotiorum* (Figures 2A and S3). At 24 h after inoculation (hai), wild‐type plants exhibited higher disease severity values, whereas transgenic Line #1 showed an 85.6% reduction in symptom development compared with the wild type, indicating enhanced resistance. Although Line #2 exhibited a marked reduction in disease severity (81.8%) compared to wild‐type plants, this difference was not statistically significant (*p* > 0.05). Likewise, Line #3 showed only an 8.4% reduction and did not differ significantly from wild‐type plants (Figure 2C).

An important observation was that transgene expression levels were not directly proportional to disease resistance. Although Line #3 exhibited the highest *CHI‐B4 like* transcript accumulation, it did not consistently show the strongest resistance phenotype. Conversely, Lines #1 and #2 displayed lower transcript levels but showed equal or greater disease suppression. Similar discrepancies between transgene expression and phenotypic performance have been reported in plant transformation studies and may be associated with differences in transgene regulation, protein accumulation, positional effects of T‐DNA insertion, or interactions with endogenous defense networks [24, 25]. These findings indicate that transcript abundance alone is not a reliable predictor of resistance intensity and that the final phenotype likely depends on the functional integration of the transgene product within the plant defense system.

Consistent with this interpretation, endochitinase activity showed a partial association with the resistance phenotype, suggesting that increased enzymatic activity contributes to, but does not solely determine, pathogen resistance. Line #1 combined elevated endochitinase activity with reduced disease severity caused by *S. sclerotiorum*. Although Line #2 exhibited the highest endochitinase activity, its resistance level was not proportionally increased, indicating that additional defense components may influence disease outcome. This hypothesis is supported by the differential expression of defense‐related genes, including *SlACO1*, *SlACS2*, *SlLoxB*, *SlLoxD*, *SlPAL5*, and *SlPR1*. Notably, *SlACO1*, *SlACS2*, and *SlPR1* showed higher transcript accumulation in Line #1, which exhibited the strongest reduction in *S. sclerotiorum* severity. These genes are associated with key defense‐related pathways involving ethylene, jasmonic acid, phenylpropanoid metabolism, and systemic acquired resistance. Thus, the superior resistance phenotype of Line #1 may result from the synergistic interaction between increased endochitinase activity and enhanced activation of multiple defense pathways, highlighting the complex regulation of resistance in *CHI‐B4 like*‐expressing plants.

Several studies have demonstrated that plant chitinases contribute to disease resistance, particularly against fungal pathogens [19, 21]. The marked reduction in *S. sclerotiorum* severity observed in Line #1 is consistent with the well‐established role of chitinases in fungal defense through the hydrolysis of chitin polymers present in fungal cell walls [17]. However, the enhanced resistance observed against Xep suggests that *CHI‐B4 like* also contributes to plant protection through broader defense mechanisms beyond its direct enzymatic activity. Similar responses have been reported in transgenic plants expressing chitinase genes, in which increased resistance was associated not only with chitin degradation but also with activation of endogenous defense pathways [19, 22]. Therefore, the resistance phenotype observed in the present study likely results from the combined effects of direct enzymatic activity and modulation of host immune responses.

Although the antifungal activity of plant chitinases has been extensively documented, evidence supporting their contribution to resistance against bacterial pathogens remains limited [20]. The significant reduction in disease severity caused by Xep observed in all three transgenic lines therefore expands the current evidence regarding the protective role of plant chitinases against bacterial diseases. Taken together, the molecular, biochemical, and phenotypic analyses indicate that *CHI‐B4 like*‐mediated resistance cannot be explained by transgene expression or endochitinase activity alone. Instead, the enhanced resistance observed in the present study appears to result from the coordinated action of increased endochitinase activity and activation of endogenous defense pathways, particularly those involving salicylic acid and ethylene signaling.

Overall, our results demonstrate that *CHI‐B4 like* overexpression enhances tomato resistance against two taxonomically distinct pathogens and provide evidence that this resistance is associated with both increased endochitinase activity and activation of endogenous defense pathways. From a breeding perspective, genes capable of conferring resistance to multiple pathogens are particularly valuable because they may reduce the need to pyramid several resistance genes targeting individual diseases. Our findings reinforce previous evidence that plant chitinases play an important role in plant immunity [26] while further demonstrating that *CHI‐B4 like*‐mediated resistance likely involves coordinated activation of multiple defense mechanisms. Together, these results highlight the potential of this gene for future biotechnological strategies aimed at improving durable and broad‐spectrum disease resistance in tomato.

## 3 Materials and Methods

### 3.1 *CHI‐B4 Like* Gene Overexpression in Tomato

The coding sequence (CDS) of the *CHI‐B4 like* (Bo4g020920), with 1,073 bp, regulated by the *Cauliflower Mosaic Virus* (CaMV 35S) promoter, was synthesized and cloned by Epoch Life Science Inc. (Sugar Land, TX, USA) into the binary vector *pBIN61* (Figure S4). This vector includes the *NPTII* gene, encoding the Neomycin Phosphotransferase II enzyme for kanamycin resistance. The resulting genetic construct, denominated pBIN61:*CHI‐B4 like*, was introduced into *Agrobacterium tumefaciens* strain EH105 using the electroporation method [27].

The transgenic plants overexpressing the *CHI‐B4 like* gene were generated using a modified methodology based on the approach proposed by Pino et al. [28]. Tomato seeds (*S. lycopersicum* L. cv. MicroTom) were germinated on MS culture medium containing half‐strength salts and B5 vitamins. The seeds were incubated at 25°C in darkness for 4 to 5 days, followed by an additional 3 days under a 16‐hour light/8‐hour dark photoperiod [29]. Subsequently, explants were prepared by transversely cutting the cotyledons and removing both the distal and proximal tips. The explants were then incubated with agitation for 15 min in liquid MS medium containing *A. tumefaciens* harboring the pBIN61:*CHI‐B4 like* vector, adjusted to an optical density of OD_600_ = 0.3 and supplemented with 100 µM acetosyringone. After this incubation, the explants were placed with the abaxial side down on plates containing a solid co‐culture medium composed of full‐strength MS salts and B5 vitamins, supplemented with 30 g/L sucrose, 5 µM 6‐benzylaminopurine (BAP), 0.1 µM indole‐3‐acetic acid (IAA), and 100 µM acetosyringone. The plates were then incubated in the dark for 48 h.

Subsequently, the explants were transferred to a solid medium for bud induction, which contained full strength MS salt and B5 vitamins, supplemented with 5 µM BAP, 0.1 µM IAA, 5 µM zeatin, 200 mg/L^−1^ cefotaxime, 100 mg/L^−1^ timentin, and 50 mg/L^−1^ kanamycin as a selection agent. The explants were retransferred to the same fresh solid selection medium every 10 days, for a total of three transfers. After this period, the shoots were excised from the buds and transferred to a solid culture medium containing full‐strength MS salts and B5 vitamins, supplemented with 30 g/L^−1^ sucrose and 50 mg/L^−1^ kanamycin to promote shoot elongation and rooting. Once elongated and rooted, the T0 transgenic plants were transferred to pots filled with a 1:1 mixture of organic substrate and vermiculite. These plants were then grown in a greenhouse to generate T2 transgenic lines.

### 3.2 Molecular Characterization of Transgenic Lines

Molecular analyses were conducted to confirm the integration and expression of the *CHI‐B4‐like* gene in transgenic tomato lines. DNA was extracted from the leaves using the CTAB method [30], and PCR amplification was performed using specific primers for the *CHI‐B4‐like* gene (Table S1), designed to amplify an 818‐base‐pair fragment. The presence of the transgene was verified by visualizing the PCR product on a 1.5% agarose gel, checking for bands corresponding to the expected size of the amplified genomic region. Positive lines were subjected to Sanger sequencing to confirm that the amplicons visualized on the agarose gel matched the genomic region of the *CHI‐B4‐like* gene.

Additionally, RT‐qPCR was performed using cDNA to quantify the expression of the *CHI‐B4‐like* transgene and to evaluate the expression of tomato defense‐related genes in the transgenic lines. For this, total RNA extraction was performed from leaves of the three transgenic lines (Line #1, #2, and #3) and the wild type, with each group represented by three individual plants (biological replicates). For RNA isolation, the TRIzol reagent (Invitrogen) was used following the manufacturer's instructions. The integrity of the RNA was confirmed by 1% electrophoresis gel agarose, and the concentration and purity (A260/A280 and A260/A230 ratios) were determined using a NanoDrop 1000 spectrophotometer (Thermo Fisher Scientific). To remove genomic DNA contaminants, the total RNA samples (4 µg per reaction) were treated with DNase I enzyme (Invitrogen), according to the manufacturer's instructions. Subsequently, cDNA synthesis was performed with GoScript Reverse Transcription System (Promega). The synthesized cDNA was diluted twenty‐times and stored at −20°C. qRT‐PCR assays were performed using gene‐specific primers for the *CHI‐B4 like* transgene to evaluate transcript accumulation in the transformed lines. Defense‐related genes associated with plant responses to phytopathogens were selected based on previous studies reported in the literature. For normalization of gene expression data, the validated reference genes Methylated histone binding protein and Small nuclear ribonucleoprotein family protein were used as endogenous controls (Table S1). The reactions were performed from cDNA of all biological replicates using Taq DNA Polymerase (Applied Biological Materials Inc.), according to the manufacturer's instructions. The melting curves of all reactions were analyzed to ensure amplification specificity.

The ΔCt values—calculated as:—*Cq* (line)—*Cq* (mean of reference genes)—were used to evaluate transgene expression levels. As the transgene was undetectable in wild‐type plants (*Cq* ≥ 40). Normality of ΔCt values was assessed using the Shapiro –Wilk test, and homogeneity of variances was verified using Levene's test. A one‑way analysis of variance (ANOVA) was performed to test for significant differences among the three transgenic lines. Subsequently, Tukey's honestly significant difference (HSD) post‑hoc test was applied for multiple pairwise comparisons. Results are presented as mean ΔCt ± standard deviation (SD). Statistically significant differences between lines are indicated by distinct letters (a or b) according to Tukey's HSD grouping, where lines sharing the same letter are not significantly different (*p* < 0.05). The statistical analyses were performed using SigmaPlot 15.0 software (Systat Software Inc., San Jose, CA, USA). Relative expression analysis of defense‐related genes was performed using the REST software based on quantification cycle (Cq) values. PCR amplification efficiencies were estimated using the Real‐Time PCR Miner online tool (http://118.190.66.83/miner/). Relative cDNA levels between treatments were calculated by REST, and statistical significance was determined using the pairwise fixed reallocation randomization test implemented in the software.

### 3.3 Protein Extraction and Enzymatic Assays

For soluble protein extraction, approximately 3 g of fresh plant material was collected from six individual plants per treatment, arranged in three biological replicates, each consisting of two pooled plants. The resulting powder was resuspended in 2 mL of 10 mM Tris‐HCl buffer, pH 8.0, supplemented with PMSF at a final concentration of 0.1 mM. The suspension was then centrifuged at 14,000 × g at 4°C for 20 min, and the clarified supernatant was used for enzymatic assays. To determine hydrolytic activity against chitin, ground solubilized chitin was used as the substrate. Briefly, 60 µL of 2% (w/v) chitin prepared in 50 mM sodium acetate buffer, pH 5.0, were mixed with 20 µL of 10‐fold diluted supernatant containing the recombinant endo‐chitinase. The reaction was carried out at 40°C for 1 h in a thermocycler. Subsequently, 120 µL of DNS reagent were added [31] the reaction mixture was boiled for 10 min. After color development, absorbance was measured spectrophotometrically at 540 nm. Results are expressed as µmol of product formed per minute per milliliter of enzyme solution. The data from biological replicates was subjected to ANOVA and subsequently analyzed using the Tukey test (*p* ≤ 0.05).

### 3.4 Plant Lines Resistance Analyses

T2 plants from three positive lines and non‐transformed (wild‐type) plants were grown in a greenhouse to evaluate the resistance of the transgenic plants to Xep and *S. sclerotiorum*. All pathogenicity assays were conducted using independent plants with biological replication. Five plants at the 3–4 leaf stage (21‐day after emergence) were inoculated with Xep T3 race strain EH 2012–22 from the working collection of the Plant Pathology Laboratory at Embrapa Hortaliças in Brasília, DF, Brazil. The inoculation was performed by spraying a bacterial suspension at approximately 5 × 10^8 ^UFC/mL^−1^ (optical density at 600 nm [OD_600_] = 0.3) until reaching the run‐off point. Following inoculation, the plants were maintained in a humid chamber for 48 h under relative humidity conditions above 90% to facilitate infection by Xep.

The inoculation of *S. sclerotiorum* (BRM 29,673), obtained from Embrapa Arroz e Feijão in Santo Antônio de Goiás, GO, Brazil was performed on plants at the 4–5 leaf stage (28 days after emergence). A mycelial disc measuring 3 mm in diameter, taken from fungal growth on Potato Dextrose Agar (PDA) after five days of incubation, was placed on leaves of five plants from each transgenic line and from the wild type. The plants were then maintained in a humid chamber for 24 h under relative humidity conditions above 90% to promote successful infection by Sclerotinia.

To quantify disease severity, the leaves inoculated with *S. sclerotiorum* from each treatment were digitized 24 hai, while the leaves from the Xep trial were digitized 12 dai. The images were analyzed using the computational program ImageJ version 1.54d [32] to determine the lesioned leaf area. Healthy and damaged regions were previously delineated using different colors, allowing the segmentation of the areas of interest by the “Color Threshold” tool. After selecting the pixels corresponding to each region, the areas were measured using the software's area analysis tools. The percentage of lesioned area was calculated as the ratio between the lesioned area and the total leaf area of each image. Data from the evaluations of *S. sclerotiorum* were initially assessed for data distribution. Since the assumptions of normality and homogeneity of variances were not met, non‐parametric analysis was performed. Differences among groups were evaluated using the Kruskal—Wallis test followed by Dunn's multiple comparison test using wild‐type plants as the control (*p* ≤ 0.05). All statistical analyses were conducted using five biological replicates per treatment. The data on bacterial spot severity obtained from independent biological replicates were subjected to ANOVA and subsequently analyzed using the Tukey test (*p* ≤ 0.05).

## 4 Conclusions

In this study, it was possible to demonstrate the role of the endochitinase *CHI‐B4‐like* in broad‐spectrum resistance. The use of the *CHI‐B4‐like* gene overexpression represents an interesting tool for increased tomato resistance to bacterial and fungal pathogens. Furthermore, this candidate gene may also be evaluated for other important tomato pathogens, offering a promising strategy for the development of multiple disease‐resistant cultivars.

## Author Contributions

**Lucas José de Sousa**: methodology, visualization, writing – review and editing, writing – original draft, investigation, software, validation, formal analysis, data curation. **Ivonaldo Reis Santos**: methodology, software, formal analysis, writing – review and editing. **Ana Clara Muniz Martins Vilela**: methodology, writing – review and editing, software, formal analysis. **Luiz Eduardo Bassay Blum**: writing – review and editing, methodology, validation, supervision, resources, project administration, data curation, investigation, visualization, conceptualization. **Ana Carolina Mendes Bezerra**: methodology, writing – review and editing. **Alice Maria Quezado‐duval**: writing – review and editing, methodology, visualization, validation, supervision, investigation, conceptualization. **Angela Mehta**: writing – review and editing, funding acquisition, investigation, conceptualization, methodology, validation, visualization, software, formal analysis, project administration, resources, supervision, data curation. **Eliane Ferreira Noronha**: conceptualization, investigation, writing – review and editing, methodology, data curation, formal analysis. **Osmundo Brilhante de Oliveira Neto**: writing – review and editing, methodology, supervision, validation. **Pedro Ricardo Vieira Hamann**: investigation, methodology, visualization, validation, writing – review and editing, formal analysis. **Glaucia Barbosa Cabral**: writing – review and editing, supervision, methodology, investigation, conceptualization.

## Conflicts of Interest

The authors declare no conflicts of interest.

## Supporting information




**Supporting File**: biot70297‐sup‐0001‐SuppMat.docx.

## Data Availability

The data that support the findings of this study are available from the corresponding author upon reasonable request.
