# Supplementary material for: Endochitinase Overexpression in Transgenic Tomato Plants Improves Resistance to Phytopathogens
Source: Biotechnol J. 2026 Aug 20;21(8):e70297. doi: 10.1002/biot.70297 (PMC13490967; doi:10.1002/biot.70297)
Supplement: Supplementary file 1 [file BIOT-21-e70297-s001.docx]

Supplementary Material

**Supplementary Figure 1.** Alignment of transgenic lines sequences with the *CHI-B4 like* and wild-type reference.

**Supplementary Figure 2.** Symptoms induced by *Xanthomonas euvesicatoria* pv. *perforans* infection at 12 days after inoculation (dai) in wild-type and transgenic plant lines.

**Supplementary Figure 3.** Symptoms induced by *Sclerotinia sclerotiorum* infection at 24 hours after inoculation (hai) in wild-type and transgenic plant lines.

**Supplementary Figure 4.** Schematic representation of the pBIN61 binary vector containing the expression cassette used for constitutive overexpression of the *BoCHB4* gene in tomato.

**Supplementary Table 1.** Primers used for PCR and RT-qPCR analysis

| **Gene** | **Access** | **Description** | **Primer Sequence 5’-3’ F/R** | **TM (ºC)** | **Reference** | **Amplicon size (bp)** | **qRT-PCR efficiency** |
| --- | --- | --- | --- | --- | --- | --- | --- |
| *CHI-B4* | Bo4g020920 | *Endochitinase* | F-ATGACCAGTTTGCTACTTTACTCA  R- TTAACAAGTGAGGTTTTCTCCAGG | 58 | (Santos et al., 2019) | 818 | - |
| *CHI-B4* | Bo4g020920 | *Endochitinase* | F-ACGCTAAAACCACATCACGA  R- GTTCCACAGTAACCAGCACT | 60 | This study | 148 | 92 |
| *SlACO1* | Solyc07g049530 | *1-Aminocyclopropane-1-Carboxylate Oxidase* | F- ATTGGCTGAGGAGTTACTTG  R- GCTTAGGACATGGTGGATAG | 60 | (Houben and Van de Poel, 2019) | 134 | 86 |
| *SlACS2* | Solyc01g095080 | *1-Aminocyclopropane-1-Carboxylate Synthase* | F- ACAAGAGGAGGAAGAGTTAGA  R- TGGGTAGTATGGTGAAGGT | 60 | (Houben and Van de Poel, 2019) | 135 | 88 |
| *SlLoxB* | Solyc01g099190 | ***Lipoxygenase B*** | F-CCTATGCCTCAAGAACTCTGCTC  R-CTCCGTCTGGATGTGGCAA | 60 | (Mariutto et al., 2011) | 90 | 87 |
| *SlLoxD* | Solyc03g122340 | ***Lipoxygenase D*** | F-TCACCAACTCGTCAACCACTG  R-ATGCATCGCGCTTAACTGC | 60 | (Mariutto et al., 2011) | 88 | 86 |
| *SlPal5* | Solyc09g007910 | ***Phenylalanine ammonia-lyase 5*** | F-CAATGGCTTCTTACTGCTCGG  R-CATCTTGGTTGTGTTGCTCAGC | 60 | (He et al., 2020) | 87 | 86 |
| *SlPR1* | Solyc09g007010 | *Pathogenesis-Related 1* | F-CAAGAGCGGGTGATTGTAA  R-CATTGGTTGGTAGCGTAGTT | 60 | (Fan et al., 2021) | 139 | 88 |
| ** PHD* | Solyc06g051420 | *Methylated histone binding* | F- ATTCGTGGCTGCTCTCTGTC  R- TAAAGGCTTCCTCCCTGTCA | 60 | (Müller et al., 2015a) | 128 | 90 |
| ** LSM7* | Solyc09g009640 | *Small nuclear ribonucleoprotein family protein* | F- GGTGGAAGACAAGTGGTTGG  R- TTCAGGGGATCATCAGCAT | 60 | (Müller et al., 2015b) | 104 | 90 |

* Primers used for gene expression normalization

**Cited references**

Fan, K.-T.; Hsu, Y.; Yeh, C.-F.; Chang, C.-H.; Chang, W.-H.; Chen, Y.-R. Quantitative proteomics reveals the dynamic regulation of the tomato proteome in response to *Phytophthora infestans*. *International Journal of Molecular Sciences*, v. 22, n. 8, p. 4174, 2021. DOI: 10.3390/ijms22084174.

He, M.; Shah Jahan, M.; Wang, Y.; Sun, J.; Shu, S.; Guo, S. Compost amendments based on vinegar residue promote tomato growth and suppress bacterial wilt caused by *Ralstonia solanacearum*. *Pathogens*, v. 9, n. 3, p. 227, 2020. DOI: 10.3390/pathogens9030227.

Houben, M.; Van de Poel, B. 1-Aminocyclopropane-1-carboxylic acid oxidase (ACO): The enzyme that makes the plant hormone ethylene. *Frontiers in Plant Science*, v. 10, p. 695, 2019. DOI: 10.3389/fpls.2019.00695.

Mariutto, M.; Duby, F.; Adam, A.; Bureau, C.; Fauconnier, M.-L.; Ongena, M.; et al. The elicitation of a systemic resistance by *Pseudomonas putida* BTP1 in tomato involves the stimulation of two lipoxygenase isoforms. *BMC Plant Biology*, v. 11, p. 29, 2011. DOI: 10.1186/1471-2229-11-29.

Müller, O. A.; Grau, J.; Thieme, S.; Prochaska, H.; Adlung, N.; Sorgatz, A.; et al. Genome-wide identification and validation of reference genes in infected tomato leaves for quantitative RT-PCR analyses. *PLoS ONE*, v. 10, n. 8, e0136499, 2015. DOI: 10.1371/journal.pone.0136499.

Santos, C.; Nogueira, F. C. S.; Domont, G. B.; Fontes, W.; Prado, G. S.; Habibi, P.; et al. Proteomic analysis and functional validation of a *Brassica oleracea* endochitinase involved in resistance to *Xanthomonas campestris*. *Frontiers in Plant Science*, v. 10, p. 414, 2019. DOI: 10.3389/fpls.2019.00414.
